# The effect of continuous adductor canal block combined with distal interspace between the popliteal artery and capsule of the posterior knee block for total knee arthroplasty: a randomized, double-blind, controlled trial

**DOI:** 10.1186/s12871-022-01712-7

**Published:** 2022-06-06

**Authors:** Chun-Guang Wang, Wen-hai Ma, Rui Liu, Ming-Yu Yang, Yang Yang, Yan-Ling Ding

**Affiliations:** 1 Department of Anesthesiology, The First Central Hospital of Baoding, Northern Greatwall Street 320#, Baoding, 071000 Hebei China; 2 Department of Orthopedics, The First Center Hospital of Baoding, Baoding, 071000 Hebei China

**Keywords:** Arthroplasty, Nerve block, Adductor canal, Popliteal artery, Capsule, Knee

## Abstract

**Background:**

The optimal analgesia for total knee arthroplasty (TKA) requires excellent analgesia while preserving muscle strength. This study aimed to determine the hypothesis that continuous adductor canal block (CACB) combined with the distal interspace between the popliteal artery and the posterior capsule of the knee (IPACK) block could effectively alleviate the pain of the posterior knee, decrease opioids consumption, and promote early recovery and discharge.

**Methods:**

Patients undergoing unilateral, primary TKA were allocated into group CACB+SHAM (receiving CACB plus sham block) or group CACB+IPACK (receiving CACB plus IPACK block). The primary outcome was cumulative opioid consumption. Secondary outcomes included the incidence of postoperative pain originated from the posterior knee, visual analogue scale (VAS) score, range of motion, ambulation distance, and satisfaction for pain management.

**Results:**

The incidence of moderate-severe pain of the posterior knee was lower in group CACB+IPACK than that of the group CACB+SHAM at 4 hours (17.1% vs. 42.8%; *p* = 0.019), 8 hours (11.4% vs. 45.7%; *p* = 0.001), and 24 hours (11.4% vs. 34.3%; *p* = 0.046) after TKA. The VAS scores of the posterior knee were lower in group CACB+IPACK than that of the group CACB+SHAM at 4 hours [2 (2) vs. 3 (2–4); *p* = 0.000], 8 hours [1 (1, 2) vs. 3 (2–4); *p* = 0.001], and 24 hours [1(0–2) vs. 2 (1–4); *p* = 0.002] after TKA. The overall VAS scores were lower in group CACB+IPACK than that of the group CACB+SHAM at 4 hours [3 (2, 3) vs. 3 (3, 4); *p* = 0.013] and 8 hours [2 (2, 3) vs. 3 (2–4); *p* = 0.032] at rest and 4 hours [3 (3, 4) vs. 4 (4, 5); *p* = 0.001], 8 hours [3 (2–4) vs. 4 (3–5); *p* = 0.000], 24 hours [2 (2, 3) vs. 3 (2–4); p = 0.001] during active flexion after TKA. The range of motion (59.11 ± 3.90 vs. 53.83 ± 5.86; *p* = 0.000) and ambulation distance (44.60 ± 4.87 vs. 40.83 ± 6.65; *p* = 0.009) were superior in group CACB+IPACK than that of the group CACB+SHAM in postoperative day 1. The satisfaction for pain management was higher in group CACB+IPACK than that of the group CACB+SHAM [9 (8, 9) vs. 8 (7–9); *p* = 0.024]. There was no difference in term of cumulative opioids consumption between group CACB+IPACK and group CACB+SHAM [120(84–135) vs. 120(75–135); *p* = 0.835].

**Conclusion:**

The combination of CACB and distal IPACK block could decrease the incidences of moderate-severe posterior knee pain, improve the postoperative pain over the first 24 hours after TKA, as well as promoting recovery of motor function. However, the opioids consumption was not decreased by adding distal IPACK to CACB.

**Trial registration:**

This study was registered at Chinese Clinical Trial Registry (ChiCTR2200059139; registration date: 26/04/2022; enrollment date: 16/11/2020; http://www.chictr.org.cn).

## Introduction

Knee Osteoarthritis, as one of the most common forms of knee disease, is widely found in the elderly population. Total knee arthroplasty (TKA) has been recognized as an effective therapy for patients with end-stage knee osteoarthritis. However, patients underwent TKA usually experience excruciating postoperative pain which decreases patient satisfaction, hampers early mobilization, prolong hospital stay, and worsen postoperative function. Therefore, anesthesiologists and surgeons are consistently seeking ways to effectively manage the postoperative pain. Recent studies found that the pain after TKA could be controlled by femoral and sciatic nerve blocks [1, 2]. However, motor never block could prevent rapid recovery, extend patients’ hospital stays.

The ideal postoperative analgesia management strategy of TKA requires not only providing adequate postoperative analgesia but also retaining the muscle strength of the limb at most. Adductor canal block (ACB) was a motor-sparing nerve block that provides an analogous analgesic effect to femoral nerve block [3–5]. However, ACB could only block the anteromedial sensory nerves of the knee joint, and approximately 72–89% of patients suffered severe postoperative pain originated from the posterior of the knee [6]. Therefore, it has become the direction of our research to do an excellent job of posterior knee analgesia. Anatomical study found that the injection of dye into the interspace between the popliteal artery and the posterior capsule of the knee (IPACK) could spread into the entire popliteal fossa and stain popliteal nerve plexus [7, 8]. Thus, it is believed that the IPACK block could block the terminal branches of the obturator and sciatic nerve. Based on these findings, we hypothesized that the IPACK block, as a motor-sparing block, could effectively alleviate the pain of the posterior knee, decrease opioids consumption, and promote early recovery and discharge. This study was designed to evaluate the analgesic effect of continuous ACB (CACB) combined with distal IPACK block for patients undergoing TKA.

## Materials and methods

The prospective randomized double-blind controlled study complied with the Declaration of Helsinki and obtained the approval from the Ethical Committee of the First Central Hospital of Baoding (NO. [2020] 092). This study was registered at Chinese Clinical Trial Registry (ChiCTR2200059139; registration date: 26/04/2022; enrollment date: 16/11/2020; http://www.chictr.org.cn/). Patients who were scheduled for primary unilateral TKA were recruited. Subjects with chronic kidney disease or cardiac insufficiency, chronic use of analgesics (daily use at least 60 mg morphine equivalents for > 4 weeks) or psychotropics, allergy to ropivacaine, contraindication to nerve block, inability to comprehend or cooperate to accomplish this study were excluded. The written informed consent was obtained from all subjects. Subjects were randomly allocated into the CACB+SHAM group (receiving CACB plus sham block) and the CACB+IPACK group (receiving CACB plus IPACK block) with a 1:1 ratio based on a computer-generated randomization sequence. Random allocation was carried out using a sealed envelope containing numbered card which was not opened until the nerve block was implemented.

Patients were educated and familiarized with visual analogue scale (VAS) score. All nerve block procedures were performed by the same senior anesthesiologist before anesthesia induction, whereas assessment was accomplished by junior anesthesiologists. Except for the nerve block team including a senior anesthesiologist and an anesthesia nurse, other participants including junior anesthesiologists participating in assessment, nurses on the floor, surgeons and patients were blinded to randomization.

### CACB

**Fig. 1:**
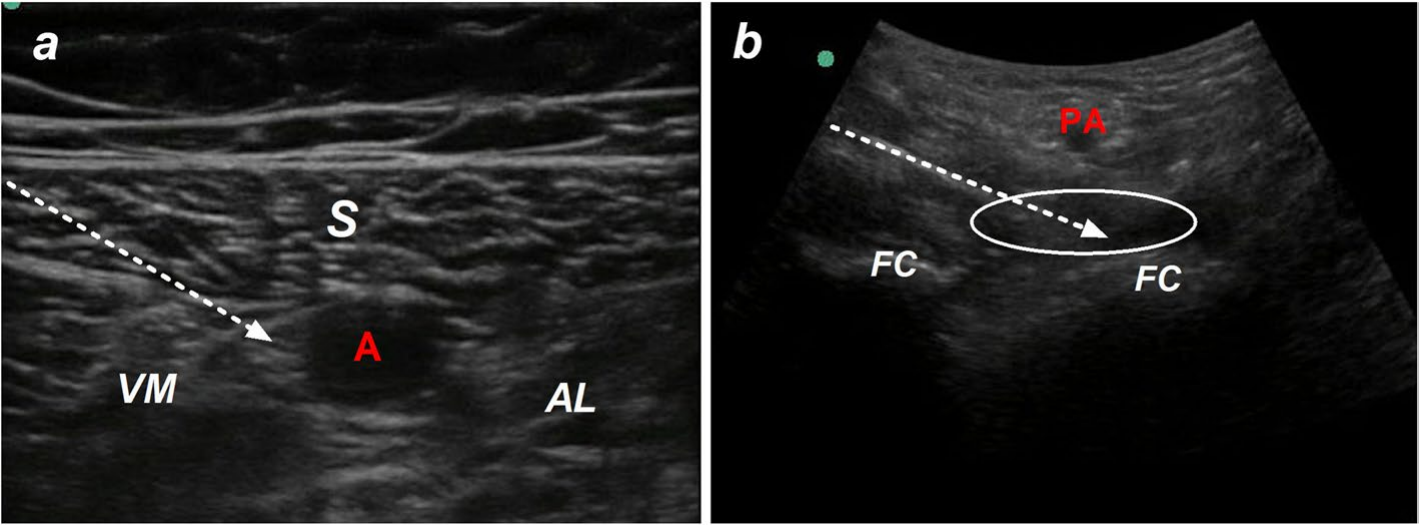
Adductor canal (**a**) and interspace between the popliteal artery and capsule of the posterior knee (**b**). S indicates sartorius muscle, VM indicates vastus medialis muscle, AL indicates adductor longus muscle, A indicates femoral artery, FC indicates femoral condyles, PA indicates popliteal artery, white arrow indicates needle of nerve block, and white ellipse indicates interspace between the popliteal artery and capsule of the posterior knee

All patients received a CACB as our previously reported (Fig. 1a). Fifteen milliliters of 0.5% ropivacaine was injected into the adductor canal after confirming the location of the catheter. Hydrodissection was used to confirm the position of the catheter. Patient-controlled nerve block analgesia (PCNA) was adopted for postoperative analgesia with 0.2% ropivacaine. The background infusion rate of PCNA was 5 ml/h, whose bolus was 5 ml, and lock-out was 30 minutes. In order to avoid local anesthetic poisoning, the maximum dose of ropivacaine was set at 600 mg/day. PCNA was discard at 72 h after TKA.

### Distal IPACK block

Patients assigned to group IPACK received a distal IPACK in a posterior approach, according to the method reported by Kampitak W et al. (Fig. 1b) [9]. In the prone position, ultrasound transducer was scanned in the popliteal crease until femoral condyle was confirmed. The needle was advanced using an in-plane approach from medial to lateral and reached the intercondylar fossa between the popliteal artery and the femoral condyles. After the needle tip was confirmed, 20 ml of 0.25% ropivacaine with 0.1 mg epinephrine was injected. Patients assigned to group SHAM underwent an IPACK with 0.9% saline subcutaneously.

### General anesthesia and postoperative analgesia

Anesthesia induction was performed after confirming the effect of nerve block. Propofol, remifentanil, sevoflurane, and cisatracurium were used for anesthesia induction and maintenance. Ventilation by laryngeal mask, end-tidal carbon dioxide was maintained at 35 to 40 mmHg. During the operation, bispectral index was maintained at 45 to 55, and the fluctuation of mean arterial pressure and heart rate were not more than ±10% of the baseline value. To reduce bleeding, tranexamic acid was administered intravenously at 0.5 h before surgery for all patients. All TKA with similar surgical steps and same prosthesis were accomplished by the same surgeon. To assess the analgesic effect of IPACK and ACB, periarticular infiltration, nonsteroidal anti-inflammatory drug(s) and paracetamol were not used in the present research. When the VAS score was more than 4, morphine, oxycodone, or hydromorphone, as a remedy, was administered.

### Outcomes

The primary outcome was cumulative opioid consumption. To facilitate comparison, opioid consumption was converted to oral morphine equivalents (OME). The following conversion equivalents were used: 5 mg morphine (PO) = 3.33 mg oxycodone (PO) = 1.25 mg hydromorphone (PO) = 1.67 mg morphine (IV/IM/SC) = 0.25 mg hydromorphone (IV) based on the recommendations of https://globalrph.com/medcalcs/opioid-pain-management-converter-advanced/. Secondary outcomes, including the incidence of postoperative moderate-severe pain originated from the posterior knee, the postoperative pain score originated from the posterior knee and the overall postoperative pain score, were assessed at 4, 8, 24, 48 and 72 h after surgery. Postoperative pain was measured by VAS (0–10; 0–3: mild pain, 4–6: moderate pain, 7–10: severe pain). The range of motion and ambulation distance were assessed in postoperative 1–3 days. The satisfaction for pain management was assessed at 72 h after surgery.

### Statistical analysis

According to Patterson ME’s research, 64 patients would have 80% power of detecting a difference at a 5% level of significance, using a confidence interval of 95% [10]. To prevent loss of power due to unanticipated dropout or protocol violations, 72 subjects were enrolled in this study.

SPSS 16.0 was used for statistical analysis. The Kolmogorov-Smirnov test was used for testing normal distribution of data. Age, height, weight, body mass index, length of surgery, range of motion and ambulation distance were shown as mean ± standard deviation and tested by *t* test. VAS score, opioids consumption and satisfaction for pain management were shown as median (interquartile range) and tested by Mann-Whitney *U* test. Gender and incidence of moderate-severe posterior knee pain were shown as number (%) and tested by χ^2^ test. *P* < 0.05 was considered statistically significant.

## Results

**Fig. 2:**
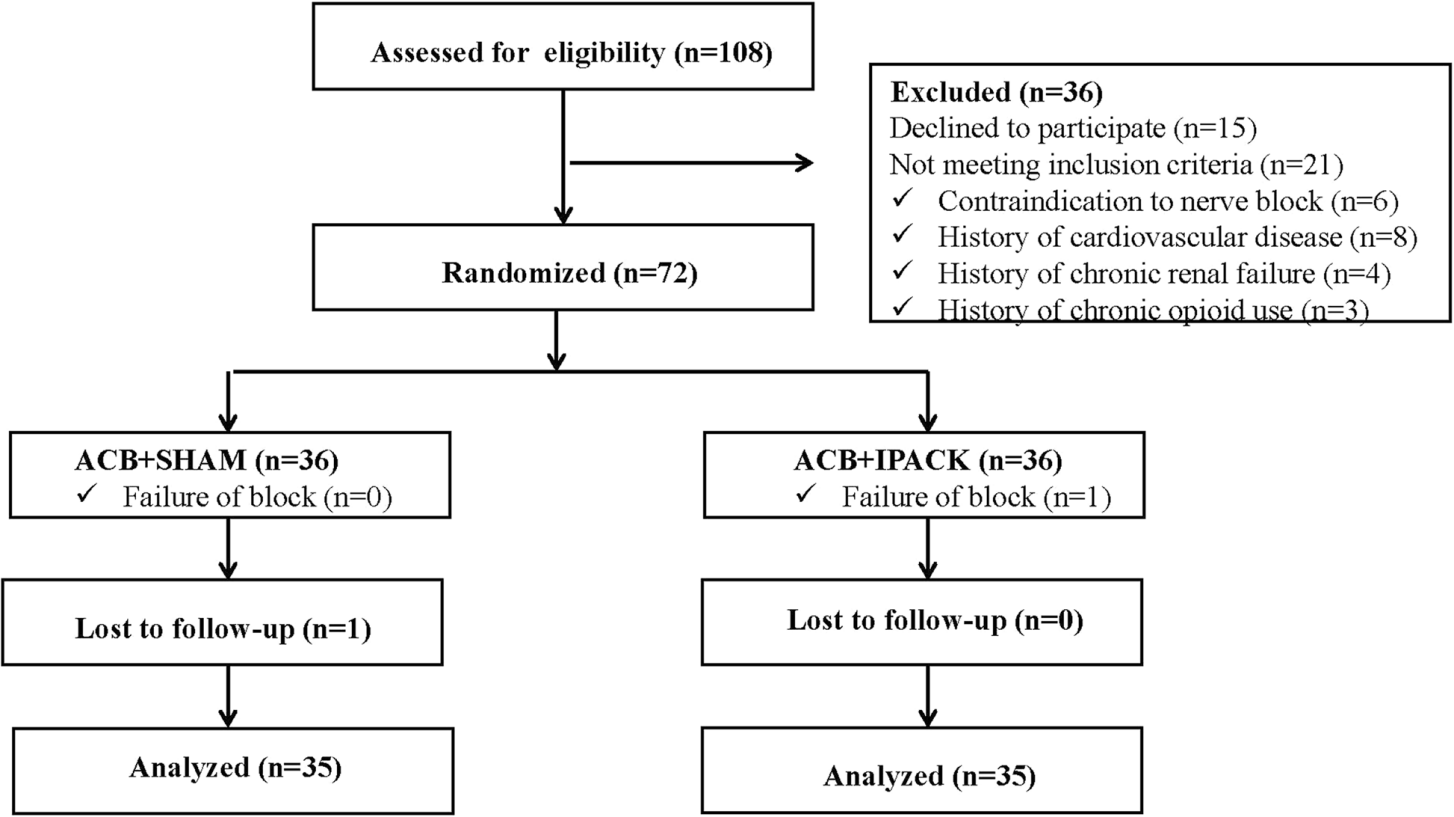
Study flow diagram. ACB indicates adductor canal block; IPACK indicates interspace between the popliteal artery and capsule of the posterior knee block; SHAM indicates sham IPACK block

Seventy-two patients were initially enrolled, and seventy of them completed the research (Fig. 2). Patient characteristics, the duration of surgery and the preoperative VAS scores were presented in Table 1. There were no significant differences in patient characteristics, the duration of surgery and the preoperative VAS scores between the two groups (Table 1; *P* > 0.05).

**Table 1:** Patient characteristics and duration of surgery

	ACB+ SHAM (*n* = 35)	ACB+ IPACK (*n* = 35)	*P* value
Age (y)	64.20 ± 5.12	66.54 ± 6.22	0.90
M/F (n)	6/29	7/28	0.76
Height (cm)	159.60 ± 7.37	157.80 ± 6.30	0.28
Weight (kg)	70.89 ± 8.70	67.71 ± 10.17	0.17
BMI (kg/m^2^)	27.82 ± 2.77	27.10 ± 3.03	0.30
Duration of surgery (min)	111.00 ± 17.27	109.86 ± 16.40	0.78
VAS at rest, preoperative	4 (3–4)	3 (3–4)	0.424
VAS during active flexion, preoperative	6 (5–6)	6 (5–6)	0.534

Data are presented as mean ± standard deviation or number

ACB indicates adductor canal block; IPACK indicates interspace between the popliteal artery and capsule of the posterior knee block; SHAM indicates sham IPACK block; M indicates male; F indicates female; BMI indicates body mass index

**Fig. 3:**
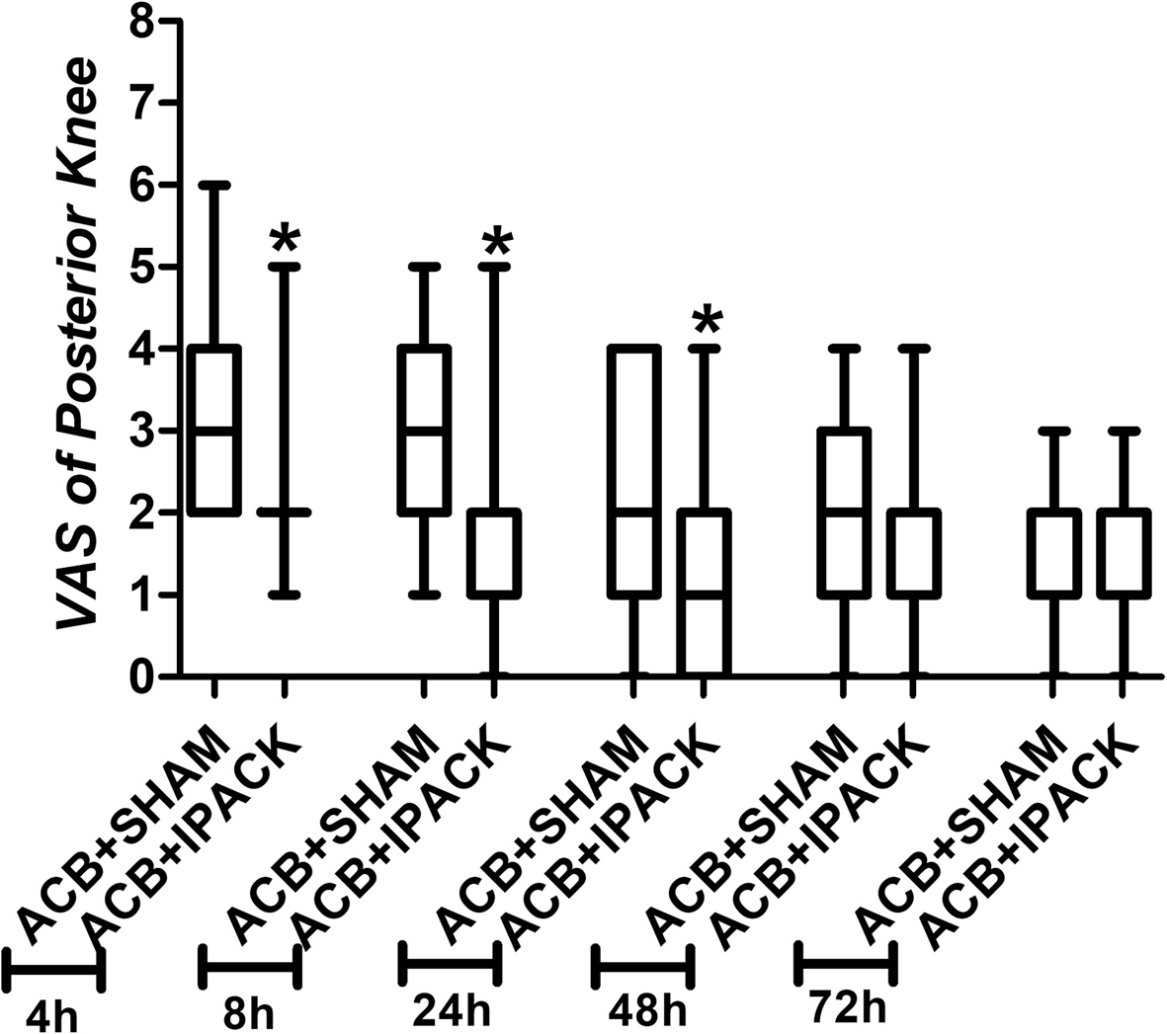
Box plots of VAS of posterior knee. VAS indicates visual analogue scale. * indicates statistically significant difference between ACB + IPACK group and ACB + SHAM group. ACB indicates adductor canal block; IPACK indicates interspace between the popliteal artery and capsule of the posterior knee block; SHAM indicates sham IPACK block

The incidence of moderate-severe pain of the posterior knee was lower in group CACB+IPACK than that of the group CACB+SHAM at 4 hours (17.1% vs. 42.8%; *p* = 0.019), 8 hours (11.4% vs. 45.7%; *p* = 0.001), and 24 hours (11.4% vs. 34.3%; *p* = 0.046) after TKA (Table 2). The VAS score of the posterior knee was lower in group CACB+IPACK than that of the group CACB+SHAM at 4 hours [2 (2) vs. 3 (2–4); *p* = 0.000], 8 hours [1 (1, 2) vs. 3 (2–4); *p* = 0.001], and 24 hours [1(0–2) vs. 2 (1–4); *p* = 0.002] after TKA (Fig. 3). The overall VAS scores were lower in group CACB+IPACK than that of the group CACB+SHAM at 4 hours [3 (2, 3) vs. 3 (3, 4); *p* = 0.013] and 8 hours [2 (2, 3) vs. 3 (2–4); *p* = 0.032] at rest and 4 hours [3 (3, 4) vs. 4 (4, 5); *p* = 0.001], 8 hours [3 (2–4) vs. 4 (3–5); *p* = 0.000], and 24 hours [2 (2, 3) vs. 3 (2–4); *p* = 0.001] during active flexion after TKA (Table 3). The range of motion (59.11 ± 3.90 vs. 53.83 ± 5.86; *p* = 0.000) and ambulation distance (44.60 ± 4.87 vs. 40.83 ± 6.65; *p* = 0.009) were superior in group CACB+IPACK than that of the group CACB+SHAM in postoperative 1 day (Table 3). The satisfaction for pain management was higher in group CACB+IPACK than that of the group CACB+SHAM [9 (8, 9) vs. 8 (7–9); *p* = 0.024] (Table 3). There was no significant difference in opioids consumption between the two groups [120(84–135) vs. 120(75–135); *p* = 0.835] (Table 3).

**Table 2:** Incidence of moderate-severe posterior knee pain

	ACB+ SHAM (*n* = 35)	ACB+ IPACK (*n* = 35)	*P* value
4 h	15 (42.8%)	6 (17.1%)	0.019
8 h	16 (45.7%)	4 (11.4%)	0.001
24 h	12 (34.3%)	4 (11.4%)	0.046
48 h	8 (22.9%)	3 (8.6%)	0.188
72 h	0 (0%)	0 (0%)	–

Data are presented as number (%)

ACB indicates adductor canal block; IPACK indicates interspace between the popliteal artery and capsule of the posterior knee block; SHAM indicates sham IPACK block

**Table 3:** Primary and Secondary Outcome

	ACB+ SHAM (*n* = 35)	ACB+ IPACK (*n* = 35)	*P* value
Primary Outcome			
Opioids Consumption (mg OME)	120 (84–135)	120 (75–135)	0.835
Secondary Outcome			
The overall VAS at rest, 4 h	3 (3–4)	3 (2–3)	0.013
The overall VAS at rest, 8 h	3 (2–4)	2 (2–3)	0.032
The overall VAS at rest, 24 h	3 (2–4)	2 (2–3)	0.060
The overall VAS at rest, 48 h	2 (1–3)	2 (1–2)	0.079
The overall VAS at rest, 72 h	2 (1–2)	2 (1–2)	0.103
The overall VAS during active flexion, 4 h	4 (4–5)	3 (3–4)	0.001
The overall VAS during active flexion, 8 h	4 (3–5)	3 (2–4)	0.000
The overall VAS during active flexion, 24 h	3 (2–4)	2 (2–3)	0.001
The overall VAS during active flexion, 48 h	3 (2–3)	2 (2–3)	0.086
The overall VAS during active flexion, 72 h	3 (2–3)	2 (2–3)	0.714
Rang of Motion (°)			
POD 1	53.83 ± 5.86	59.11 ± 3.90	0.000
POD 2	67.20 ± 5.89	67.09 ± 5.57	0.934
POD 3	97.00 ± 3.94	97.80 ± 5.58	0.504
Ambulation Distance (feet)			
POD 1	40.83 ± 6.65	44.60 ± 4.87	0.009
POD 2	66.49 ± 4.22	68.46 ± 4.85	0.074
POD 3	96.26 ± 4.95	96.48 ± 3.59	0.826
Satisfaction for pain management	8 (7–9)	9 (8–9)	0.024

Data are presented as mean ± standard deviation or median (interquartile range)

ACB indicates adductor canal block; IPACK indicates interspace between the popliteal artery and capsule of the posterior knee block; SHAM indicates sham IPACK block; POD indicates postoperative day; OME, oral morphine equivalent

## Discussion

The study was to observe the analgesic effect and early functional rehabilitation of CACB combined with distal IPACK for patients undergoing TKA. Patients who received distal IPACK combined with CACB for postoperative analgesia had lower incidences of moderate-severe posterior knee pain and lower pain score over 24 hours after the operation. Meantime, CACB combined with distal IPACK could improve the overall VAS score. Moreover, the addition of distal IPACK could facilitate patients’ early activity and improve patients’ satisfaction with pain management. However, postoperative opioids consumption was not decreased by CACB combined with IPACK.

The optimal analgesia for TKA requires excellent analgesia while preserving muscle strength. ACB was a motor-sparing nerve block for postoperative analgesia following TKA [11–13]. However, most patients still complained of postoperative pain originated from the posterior knee. Based on the anatomy evidence, the saphenous nerve, the nerve to the vastus medialis muscle and the lateral and medial femoral cutaneous nerves co-innervate sensation of the anterior knee [14–16]. Sensation of the posterior knee is co-innervated by the tibial nerve, the posterior branch of the obturator nerve, and the common peroneal nerve [14–16]. Recently research found that sciatic nerve block or selective tibial nerve block, as an analgesic supplement to ACB, could address pain originated from the posterior knee [17, 18]. Unfortunately, both sciatic nerve block and selective tibial nerve block could weaken muscle strength of lower limb, lead to foot drop, and affect early rehabilitation. Anatomical research found that the injection of dye into IPACK could spread to the entire popliteal fossa, and stain popliteal nerve plexus, which consist of the terminal branches of posterior branch of the obturator nerve and tibial nerve [7, 8]. In the present research, patients who received distal IPACK combined with CACB for postoperative analgesia had a lower incidence of moderate-severe posterior knee pain and lower posterior knee pain scores over 24 hours after the operation. These findings suggested distal IPACK could solve posterior knee pain, which was consistent with Kampitak’s results [9]. Kampitak and her colleague found that, with 20 mL of 0.25% levobupivacaine with 1:200000 epinephrine, distal IPACK could provide reliable analgesic effect for posterior knee pain during the 24-hour postoperative period, which was equivalent to tibial nerve block [9]. However, a recent study found that, with 20 mL of 0.5% ropivacaine, IPACK combined with ACB and multimodal analgesia could decreased the incidences of posterior knee pain at 6 hours after TKA [19]. The reason of difference in analgesic time of above studies might be explained by whether the local anesthetic contains adrenaline and application of multimodal analgesia. Furthermore, in this study, CACB combined with distal IPACK improved overall VAS score over 24 hours after surgery, which was consistent with Sankineani’s findings [20]. Sankineani et al. found that with 15 mL of 0.2% ropivacaine, IPACK combined with ACB analgesia could decrease the VAS score over 48 hours after surgery than ACB alone [20]. However, Ochroch et al. found that the reduction of pain score was not clinically significant by the addition of proximal IPACK to ACB [19]. Ochroch et al. carried out a proximal IPACK combined with ACB and multimodal analgesia [19], while, a distal IPACK combined with ACB without multimodal analgesia was implemented in this study. Tran et al. found that, compared with the proximal injection technique, inferior branches of the tibial nerve and the posterior branch of the common fibular nerve was stained more easily with the distal injection technique, which indicated that pain originated from the posterior knee was better controlled by the distal injection technique [7]. This hypothesis was confirmed by clinical research reported by Kampitak et al. [9] However, the improvement of postoperative pain didn’t translate to opioid consumption in the present study, which was consistent with some previous findings [10, 19]. Ochroch et al. found that IPACK block could not reduce opioids consumption over 48 hours after surgery [19]. Patterson et al. found that postoperative consumption of opioids was not decreased by the addition of IPACK to ACB [10]. However, Kim DH reported that the consumption of opioid was decreased by the combination of ACB, IPACK and periarticular injection over the first 24 hours after surgery [21]. Coincidentally, a systematic review and meta-analysis indicated that IPACK block combined with ACB could reduce opioids consumption over the first 24 hours after surgery [22]. Interestingly, a recent systematic review and meta-analysis by Nasir H et al. reported that IPACK block combined with ACB and periarticular infiltration could not improve analgesic effect after TKA. In the absence of periarticular infiltration, IPACK block combined with ACB could improve postoperative pain over the first 24 hours after surgery and enhances functional recovery [23]. It is possible that the difference is due to diversity pathways of relief postoperative pain applied in different institutions. Moreover, in this study, the commensurate opioids consumption between the two group might be due to the advanced in years of patients, their low baseline opioid consumption and the criterion of our institution to prescribe opioids base on its adverse reaction in elderly.

In this study, the combination of CACB and distal IPACK block could improve range of motion in postoperative day 1 and ambulation distance in postoperative day 1, which were consistent with Eccles’s findings [24]. The injection site of the distal injection technique is at the level of the femoral condyle in the distal portion of the popliteal fossa, where the common peroneal nerve runs superficially and away from the posterior capsule, and separates from the tibial nerve completely [9]. The injection site is far away from the trunk of common peroneal nerve and tibial nerve, so the motor function was preserved to a greatest extent [9]. Sreckovic et al. found that IPACK with the proximal injection technique could cause motor deficits, including foot drop, which probably due to the injection site of the proximal injection technique is at the level of femoral shaft [25]. At that level, common peroneal nerve is not completely branched out from the trunk of sciatic nerve. Local anesthetics could spread proximally to the trunk of common peroneal nerve, resulting foot drop. However, foot drop was not found in our study.

There are several limitations to this study. First, the sample size of the study is relatively small, therefore, the outcome may have some limitations. To a certain degree, it is possible that some undesirable complication of IPACK may be neglected, such as bleeding, infection, falls in-hospital and etc. Second, to assess the analgesic effect of IPACK combined with CACB, multimodal analgesic strategy was not implemented in the present research, which might cause insufficient analgesia. Third, the patients were followed for only 72 hours after surgery. In the further, more studies are needed to assess the effect of IPACK block on long-term analgesia and functional exercise after TKA.

It is concluded that the combination of CACB and distal IPACK block could decrease the incidences of moderate-severe posterior knee pain, improve the postoperative pain over the first 24 hours for after TKA, as well as promoting recovery of motor function. However, the opioids consumption was not decreased by the addition of IPACK to CACB.

## Data Availability

The datasets generated and/or analyzed during the current study are not publicly available due as a part of a series of studies which have not been completed, but are available from the corresponding author on reasonable request.

## Abbreviations

TKA: Total knee arthroplasty
CACB: Continuous adductor canal block
IPACK: Interspace between the popliteal artery and the posterior capsule of the knee
VAS: Visual analogue scale
PCNA: Patient-controlled nerve block analgesia
